# Pancreatic duct size and gland texture are associated with pancreatic fistula after pancreaticoduodenectomy but not after distal pancreatectomy

**DOI:** 10.1371/journal.pone.0203841

**Published:** 2018-09-13

**Authors:** Allison N. Martin, Sowmya Narayanan, Florence E. Turrentine, Todd W. Bauer, Reid B. Adams, Victor M. Zaydfudim

**Affiliations:** 1 Department of Surgery, University of Virginia, Charlottesville, VA, United States of America; 2 Surgical Outcomes Research Center, University of Virginia, Charlottesville, VA, United States of America; 3 Section of Hepatobiliary and Pancreatic Surgery, University of Virginia, Charlottesville, VA, United States of America; University of Florida, UNITED STATES

## Abstract

**Background:**

Pancreatic fistula remains a morbid complication after pancreatectomy. Since the proposed mechanism of pancreatic fistula is different between pancreaticoduodenectomy and distal pancreatectomy, we hypothesized that pancreatic gland texture and duct size are not associated with pancreatic fistula after distal pancreatectomy.

**Methods:**

All patients ≥18 years in the 2014–15 American College of Surgeons National Surgical Quality Improvement Program (ACS NSQIP) targeted pancreatectomy dataset were linked with the ACS NSQIP Public Use File (PUF). Pancreatic duct size (<3 mm, 3–6 mm, >6 mm) and pancreatic gland texture (hard, intermediate, soft) were categorized. Separate multivariable analyses were performed to evaluate associations between pancreatic duct size and gland texture after pancreaticoduodenectomy and distal pancreatectomy.

**Results:**

A total of 9366 patients underwent pancreaticoduodenectomy or distal pancreatectomy during the study period. Proportion of pancreatic fistula was similar after distal pancreatectomy (606 of 3132, 19.4%) and pancreaticoduodenectomy (1163 of 6335, 18.4%, p = 0.245). Both pancreatic gland texture and duct size were significantly associated with pancreatic fistula after pancreaticoduodenectomy (p<0.001). However, there was no association between pancreatic fistula and gland texture or duct size (all p≥0.169) after distal pancreatectomy. Operative approach (minimally invasive versus open) was not associated with pancreatic fistula after distal pancreatectomy (p = 0.626). Patients with pancreatic fistula after distal pancreatectomy had increased rate of postoperative complications including longer length of stay, higher rates of readmission and reoperation compared to patients who did not have a pancreatic fistula (all p<0.001).

**Conclusions:**

Unlike among patients who had pancreaticoduodenectomy, pancreatic gland texture and duct size are not associated with development of pancreatic fistula following distal pancreatectomy. Other clinical factors should be considered in this patient population.

## Introduction

Despite improvements in surgical technique and perioperative care, complications after pancreatectomy remain high [1–3]. The most significant technical cause of post-pancreatectomy morbidity is the formation of pancreatic fistulas, which are estimated to occur in up to 10–20% of pancreatic resections. Leakage of exocrine pancreatic juice is associated with additional pancreatectomy-specific complications such as hemorrhage and delayed gastric emptying leading to increases in length of hospital stay, reoperations, and readmissions [4–9].

Risk factors associated with pancreatic fistula after pancreaticoduodenectomy have been well established. A number of studies describing the fistula risk score have identified and validated both small pancreatic duct size and soft gland texture as the leading risk factors for pancreatic leak following pancreaticoduodenectomy [10–12]. The utility of these two pancreas-specific factors in predicting pancreatic fistula after distal pancreatectomy is not yet known.

It is likely that factors associated with pathogenesis of pancreatic fistula after pancreaticoduodenectomy and distal pancreatectomy are different. The objectives of this study were to 1) compare effects of pancreatic duct size and gland texture on development of pancreatic fistula after pancreaticoduodenectomy and distal pancreatectomy, and 2) estimate effects of clinically meaningful perioperative variables on development of pancreatic fistula after distal pancreatectomy.

## Methods

### Patient selection and variable definitions

The American College of Surgeons National Surgical Quality Improvement Program (ACS NSQIP) is a de-identified, independently collected, Health Insurance Portability and Accountability Act (HIPAA) compliant dataset that includes patient-level data from a nationwide cohort of participating hospitals. A pancreatectomy-specific targeted module was released in 2014. A combined 2014 and 2015 ACS NSQIP Participant Use File (PUF) and ACS NSQIP pancreatectomy targeted linked dataset was created and used for this retrospective cohort study. The University of Virginia Institutional Review Board (UVA IRB) for Health Sciences Research (HSR) has designated the ACS NSQIP PUF and targeted datasets as a publically available de-identified data exempt from formal IRB review.

All patients ≥18 years of age who had pancreaticoduodenectomy with pancreatic duct reconstruction (Current Procedural Terminology (CPT) codes 48150 and 48153) and distal pancreatectomy (CPT 48140) were abstracted from the linked NSQIP PUF and targeted pancreatectomy datasets. All patients who had pancreatic resection for chronic pancreatitis and patients who had incidental distal pancreatectomy for primary splenic pathology were excluded. A total of 110 patients had a missing pancreatic fistula variable; these patients were also excluded. The primary outcome was postoperative pancreatic fistula, defined according to the International Study Group on Pancreatic Fistula (ISGPF) definition [13,14]. ACS NSQIP and ISGPF definitions of pancreatic fistula are congruous. Pancreatic fistula is defined as either persistent drainage of amylase-rich fluid with an amylase level of three times the normal serum amylase activity on or after postoperative day number three and one of the following criteria: drain continued longer than seven days, requirement for percutaneous drainage or reoperation. Alternatively, pancreatic fistula is defined as a clinical diagnosis by an attending surgeon in association with one of the following criteria: drain continued longer than seven days, spontaneous wound drainage (i.e., if no drain is present), requirement for percutaneous drainage or reoperation.

The two primary independent variables tested in the study were pancreatic duct size and pancreatic gland texture. Pancreatic duct size was categorized as small <3 mm, medium 3–6 mm, or large >6 mm. Pancreatic gland texture was categorized as soft, intermediate, or hard. Other demographic and clinical variables included age, sex, race/ethnicity (i.e., white, black/African-American, Hispanic, other), smoking status, presence of diabetes, postoperative amylase level (units/liter), American Society of Anesthesiologists (ASA) class, operative duration, perioperative blood transfusion within 72 hours, surgical approach (open versus minimally invasive [laparoscopic and robotic]), intraoperative surgical site drain placement, and subsequent postoperative percutaneous drain insertion. Indication for operation was categorized as malignant or non-malignant using the final pathologic diagnosis as defined by the International Classification of Diseases, 9th revision and 10th revision (ICD-9, ICD-10) codes (summarized in **Appendix A**). In addition to pancreatic fistula, other metrics of postoperative outcomes included reoperation, readmission, and death defined using the standard ACS NSQIP definitions based on their occurrence relative to the index operation.

### Statistical analysis

Differences in distribution of categorical variables were assessed using chi-square or Fisher’s exact text, where appropriate, and reported as frequencies with percentages; continuous variables were reported as median with interquartile range (IQR) and compared using the Wilcoxon rank-sum test.

Three separate multivariable models were developed to estimate the effects of pancreatic duct size and pancreatic gland texture on pancreatic fistula after pancreaticoduodenectomy and distal pancreatectomy. Age, sex, body mass index (BMI), diagnosis, operative time, pancreatic duct size, and pancreatic gland texture were included in each multivariable model. Missing data in pancreatic duct size and pancreatic gland texture occurred in the targeted pancreatectomy dataset with the following frequencies: pancreatic duct size in pancreaticoduodenectomy cohort 23%; pancreatic duct size in distal pancreatectomy cohort 76%; pancreatic gland texture in pancreaticoduodenectomy cohort 27%; pancreatic gland texture in distal pancreatectomy cohort 61%.

There were no clinically meaningful differences in reporting of pancreatic duct or gland texture parameters among patients who did or did not develop a fistula. Missing data within multivariable models was addressed using two statistical methods: 1) missing variables within each category were assigned as “unknown” and included as an additional covariate in the multivariable model, 2) for distal pancreatectomy cohort only, given clinical likelihood, all patients with missing covariates were assigned to have normal pancreatic gland parameters at the presumed pancreatic transection margin: small <3 mm pancreatic duct and soft gland texture. All data management and statistical analysis was performed using STATA software version 14.2 (StataCorp LP, College Station, TX).

## Results

### Patient demographics and postoperative pancreatic fistula

A total of 9467 patients, median age 65 (IQR 56–72), were included in the study. A majority of the patients were white (74.4%) and non-smokers (82.7%). Sex was equally divided (50.3% male). A majority of the patients had an open operation (78.2%) and malignant indication for resection (72.3%). Surgical drains were used in 86.6% of patients during index resection.

Approximately two-thirds of patients underwent pancreaticoduodenectomy (n = 6410) and one-third underwent distal pancreatectomy (n = 3132). Overall 30-day mortality rate after pancreaticoduodenectomy was 2.4%, which is greater than the 1.2% 30-day mortality for patients who underwent distal pancreatectomy. A total of 1163 patients (18.4%) developed a pancreatic fistula after pancreaticoduodenectomy; 606 patients (19.3%) developed a pancreatic fistula after distal pancreatectomy.

Bivariable analysis compared clinical characteristics and postoperative outcomes by presence of postoperative pancreatic fistula stratified by type of operation: pancreaticoduodenectomy (**Table 1**) and distal pancreatectomy (**Table 2**). Among patients who had pancreaticoduodenectomy, pancreatic fistula was associated with small duct size, soft gland texture, non-malignant diagnosis, and longer operative time (all p<0.001). Among patients who had distal pancreatectomy, pancreatic fistula was associated with longer operative time (median = 235 [IQR 181–309] versus median = 206 [IQR 152–277], p< 0.001) and higher proportion of perioperative blood transfusion (17.8% versus 12.2%, p<0.001). Pancreatic duct size and gland texture were not associated with pancreatic leak after distal pancreatectomy (both p≥0.232).

**Table 1:** Characteristics among patients with and without pancreatic Fistula: Pancreaticoduodenectomy (n = 6335).

	Pancreatic Fistula (n = 1163)	No Fistula (n = 5172)	p-value
Age	65 (57–72)	66 (58–73)	0.035
Male Sex	685 (58.9)	2716 (52.5)	<0.001
BMI	28.0 (24.7–32.2)	26.3 (23.1–29.9)	<0.001
Race/Ethnicity			0.108
White	887 (83.8)	3881 (81.8)	
Black	74 (7.0)	435 (9.2)	
Hispanic	55 (5.2)	217 (4.6)	
Other	43 (4.1)	210 (4.4)	
Surgical Approach			0.083
Open	1057 (90.9)	4779 (92.4)	
Minimally Invasive	106 (9.1)	393 (7.6)	
Malignant Diagnosis	857 (73.7)	4274 (82.6)	<0.001
Operative time	373 (304–454)	356 (277–441)	<0.001
Diabetes	239 (20.6)	1323 (25.6)	<0.001
Smoking	186 (16.0)	937 (18.1)	0.087
Duct Size			<0.001
<3 mm	394 (33.9)	1099 (21.3)	
3–6 mm	379 (32.6)	2156 (41.7)	
>6 mm	80 (6.9)	792 (15.3)	
Missing	310 (26.7)	1125 (21.8)	
Gland Texture			<0.001
Hard	183 (15.7)	1805 (34.9)	
Intermediate	47 (4.0)	397 (7.7)	
Soft	595 (51.2)	1607 (31.1)	
Missing	338 (29.1)	1363 (26.4)	
Surgical Drain	1087 (93.5)	4446 (86.1)	<0.001
Percutaneous Drain	486 (58.1)	366 (12.3)	<0.001
Peak Amylase POD#1	2755 (988–9408)	185 (29–1330)	<0.001
Peak Amylase POD#2–30	3214 (571–13780)	30 (10–177)	<0.001
Transfusion within 72 hrs	244 (21.0)	1037 (20.1)	0.476
Length of Stay	14 (9–21)	8 (6–12)	<0.001
Reoperation	139 (12.0)	214 (4.1)	<0.001
Readmission	297 (25.6)	787 (15.2)	<0.001
Death	45 (3.9)	96 (1.9)	<0.001

IQR: interquartile range; BMI: body mass index; POD: postoperative day

Continuous variables are expressed as median (IQR) and categorical variables are expressed as *n* (%).

**Table 2:** Characteristics among patients with and without pancreatic fistula: Distal Pancreatectomy (n = 3132).

	Pancreatic Fistula (n = 606)	No Fistula (n = 2526)	p-value
Age	62 (52–69)	63 (53–72)	0.002
Male Sex	303 (50)	1046 (41.4)	<0.001
BMI	27.6 (24.0–32.0)	29.1 (24.8–33.7)	<0.001
Race/Ethnicity			0.636
White	443 (80.4)	1835 (79.6)	
Black	56 (10.2)	251 (10.9)	
Hispanic	32 (5.8)	115 (5.0)	
Other	20 (3.6)	105 (4.6)	
Surgical Approach			0.626
Open	298 (49.2)	1270 (50.3)	
Minimally Invasive	308 (50.8)	1256 (49.7)	
Malignant Diagnosis	337 (55.6)	1381 (54.7)	0.677
Operative time	235 (181–309)	206 (152–277)	<0.001
Diabetes	132 (21.8)	613 (24.3)	0.197
Smoking	120 (19.8)	400 (15.8)	0.018
Duct Size			0.764
<3 mm	80 (13.2)	347 (13.7)	
3–6 mm	47 (7.8)	167 (6.6)	
>6 mm	20 (3.3)	78 (3.1)	
Missing	459 (75.7)	1934 (80.8)	
Gland Texture			0.232
Hard	69 (11.4)	244 (9.7)	
Intermediate	25 (4.1)	82 (3.3)	
Soft	140 (23.1)	659 (26.1)	
Missing	372 (61.4)	1541 (61.0)	
Surgical Drain	569 (93.9)	2092 (83.0)	<0.001
Percutaneous Drain	210 (35.1)	150 (6.1)	<0.001
Peak Amylase POD#1	4158 (1191–8230)	1300 (233–3845)	<0.001
Peak Amylase POD#2–30	4207 (1159–15815)	111 (32–575)	<0.001
Transfusion within 72 hrs	108 (17.8)	308 (12.2)	<0.001
Length of Stay	6 (5–10)	6 (4–7)	<0.001
Reoperation	38 (6.3)	74 (2.9)	<0.001
Readmission	229 (37.8)	307 (12.2)	<0.001
Death	8 (1.3)	28 (1.1)	0.661

IQR: interquartile range; BMI: body mass index; POD: postoperative day

Continuous variables are expressed as median (IQR) and categorical variables are expressed as *n* (%).

Minimally invasive pancreaticoduodenectomy was performed in 9.1% of patients who had pancreatic fistula compared to 7.6% of patients who did not have a fistula (p = 0.083). Minimally invasive distal pancreatectomy was not associated with pancreatic fistula (p = 0.626). Surgical drains and percutaneous drainage in postoperative period were more common in patients with pancreatic fistula (all p<0.001). Reoperation and readmission were associated with pancreatic fistula after both pancreaticoduodenectomy and distal pancreatectomy (all p<0.001). Mortality was greater in patients who had pancreatic fistula after pancreaticoduodenectomy (3.9% versus 1.9%, p<0.001). There was no difference in mortality among patients who did or did not have pancreatic fistula after distal pancreatectomy (1.3% versus 1.1%, p = 0.661).

### Multivariable analyses

Both pancreatic duct size and gland texture were independently associated with pancreatic fistula after pancreaticoduodenectomy (both p<0.001, **Table 3**). Medium (OR = 0.63, 95% CI: 0.53–0.74) and large (OR = 0.41, 95% CI: 0.31–0.54) pancreatic duct sizes were associated with lesser likelihood of pancreatic fistula after pancreaticoduodenectomy. Soft pancreatic gland texture (OR = 3.00, 95% CI: 2.49–3.62) was independently associated with greater likelihood of pancreatic fistula.

**Table 3:** Pancreaticoduodenectomy: Multivariable logistic regression model results for association with pancreatic fistula.

	Number	Percent				
Total cases	6335	100.00	Odds Ratio	Odds Ratio95% ci min	Odds Ratio 95% ci max	P-value
Pancreatic fistula	1163	18.36				
Duct Size						<0.001
< 3 mm	1493	23.57	reference			
3–6 mm	2535	40.02	0.63	0.53	0.74	<0.001
> 6 mm	872	13.76	0.41	0.31	0.54	<0.001
Unknown duct size	1435	22.65	0.90	0.73	1.10	0.287
Gland texture						<0.001
Hard	1988	31.38	reference			
Intermediate	444	7.01	1.10	0.78	1.55	0.584
Soft	2202	34.76	3.00	2.49	3.62	<0.001
Unknown gland texture	1701	26.85	2.01	1.62	1.62	<0.001

In contrast, neither pancreatic duct size, nor pancreatic gland texture were associated with pancreatic fistula after distal pancreatectomy (all p≥0.169). Two separate multivariable models summarize associations between pancreatic remnant characteristics and pancreatic fistula after distal pancreatectomy. There were no associations between pancreatic characteristics and pancreatic fistula when missing data were summarized as an “unknown” covariate (all p≥0.190, **Table 4**). There were also no associations between pancreatic characteristics and pancreatic fistula when missing data were all imputed to have normal parameters for pancreatic remnant—small duct and soft gland texture (all p≥0.169, **Table 5**). Comparisons between patients with missing and available pancreatic duct size and gland texture variables are summarized in supplementary S1 and S2 Tables, respectively. There were no differences in preoperative diagnosis, or major postoperative complications including proportions of pancreatic fistula, need for percutaneous drains, transfusions, reoperations, readmissions, or deaths (all p≥0.103).

**Table 4:** Distal pancreatectomy: Multivariable logistic regression model results for association with pancreatic fistula.

	Number	Percent					
Total cases	3132	100.00	Odds Ratio	Odds Ratio 95% ci min	Odds Ratio 95% ci max	P-value	
Pancreatic fistula	606	19.35					
Duct Size							
< 3 mm	427	13.63	reference				
3–6 mm	214	6.83	1.32	0.87	2.00	0.190	
> 6 mm	98	3.13	1.26	0.72	2.20	0.421	
Unknown duct size	2393	76.40	1.01	0.76	1.34	0.970	
Gland texture							
Hard	313	9.99	reference				
Intermediate	107	3.42	1.08	0.64	1.82	0.708	
Soft	799	25.51	0.75	0.54	1.04	0.255	
Unknown gland texture	1913	61.08	0.87	0.65	1.17	0.692	

**Table 5:** Distal pancreatectomy: Multivariable logistic regression model (Imputation of <3 for all unknown duct size and soft pancreas for all unknown gland texture).

	Number	Percent					
Total cases	3132	100.00	Odds Ratio	Odds Ratio 95% ci min	Odds Ratio 95% ci max	P-value	
Pancreatic fistula	606	19.35					
Duct Size							
< 3 mm	2820	90.04	reference				
3–6 mm	214	6.83	1.27	0.90	1.80	0.169	
> 6 mm	98	3.13	1.23	0.74	2.05	0.428	
Gland texture							
Hard	313	9.99	reference				
Intermediate	107	3.42	1.11	0.65	1.88	0.707	
Soft	2730	87.16	0.90	0.68	1.21	0.498	

## Discussion

Pancreatic gland texture and duct size are not associated with development of pancreatic leak following distal pancreatectomy. Both pancreatic duct size and gland texture have a significant association with fistula formation after pancreaticoduodenectomy. The mechanism of leak and fistula formation from the pancreatic remnant are different after pancreaticoduodenectomy and distal pancreatectomy. Leak after pancreaticoduodenectomy has been attributed to technical limitations of pancreatico-intestinal anastomosis. Indeed, the pancreatic fistula risk score [10–12] incorporates both pancreatic duct size and gland texture in a validated risk calculation tool which has been used to improve specific perioperative decision-making strategies [15], as well as risk assessment of postoperative outcomes [16]. The current study evaluates a national dataset of pancreatic surgery data and expands on the recently published multi-institutional data that also examined risk factors associated with fistula development after distal pancreatectomy [17].

Pancreatic leak and fistula formation after distal pancreatectomy, however, is likely a result of functional distal obstruction by the sphincter of Oddi complex at the ampulla, which increases intraductal pressure and potentiates pancreatic stump leakage [18–20]. Previous attempts to mitigate pancreatic fistula after distal pancreatectomy focused on closure of the pancreatic stump. Multiple published prospective trials compared various management strategies of pancreatic stump closure. There were no observed differences in pancreatic fistula between hand-sewn closure versus stapler closure technique [21] or between remnant pancreatojejunostomy versus stapler closure technique [22]. A number of other adjuncts to pancreatic stump closure such as sealants, mesh reinforcement, and soft tissue pedicle flaps have been investigated, many with equivocal results [23–28]. The most recent randomized control trial of polyglycolic acid mesh reinforcement did not demonstrate a reduction in overall occurrence of pancreatic fistula after distal pancreatectomy; however, clinically relevant pancreatic fistulas were more common in patients without mesh reinforcement [29]. Ligamentum teres vascularized pedicle flaps have also been promising [30,31]; however, recent randomized controlled trial including 152 patients failed to demonstrate a reduction in pancreatic fistula after distal pancreatectomy with coverage of the transection margin by a vascularized flap [32].

Additionally, recent techniques for mitigation of pancreatic fistula after distal pancreatectomy have also focused on decreasing the pressure gradient at the sphincter of Oddi. Single institution studies with retrospective control groups appeared to demonstrate a decrease in rates of pancreatic fistula after distal pancreatectomy with use of transampullary stenting [33,34]. However, a subsequent randomized controlled trial of prophylactic transpapillary pancreatic duct stenting did not demonstrate a reduction in pancreatic fistulae among patients with preoperative ductal decompression [35]. Definitive conclusions from this single published randomized prospective trial are difficult; 23 of 53 patients who had pancreatic resection had postoperative pancreatic fistula. As such, the trial is both under-powered and the overall proportion of pancreatic fistula is high. Other strategies for decreasing the pressure gradient at the sphincter of Oddi have included preoperative injection of botulinum toxin [19] and attempted avoidance of systemic-acting opioids [18].

The overall proportions of pancreatic fistula in this study are 18.4% after pancreaticoduodenectomy and 19.3% after distal pancreatectomy. These ACS NSQIP targeted pancreatectomy fistula rates for pancreaticoduodenectomy are higher than reported single-center pancreatic fistula estimates, but are similar to the proportions from the Pancreatectomy Demonstration Project [36,37]. The ACS NSQIP targeted dataset proportion of pancreatic fistula after distal pancreatectomy is similar to the estimates from single institution studies.

Preoperative factors associated with pancreatic fistula after distal pancreatectomy in this patient population include male sex, lower BMI (although median BMI in both groups was in the 25.0–29.9 category characterized as overweight), longer operative time, and tobacco use. Male sex and tobacco use were identified previously as preoperative factors associated with pancreatic fistula after distal pancreatectomy [38]. Longer operative time could be a surrogate for more challenging operation, but further postulation is difficult. There was no difference between proportions of pancreatic fistula after distal pancreatectomy between patients who had minimally invasive or open resection. Method of pancreatic gland transection, pancreatic stump coverage adjuncts such as soft tissue flaps or mesh, as well as potential use of pre-resection pancreatic duct stents are not included in ACS NSQIP targeted pancreatectomy dataset and could not be examined in this study. A recently published analysis using granular data on over 2000 patients from 10 institutions similarly failed to demonstrate clinically relevant impact of pancreatic gland characteristics on pancreatic fistula after distal pancreatectomy [17]. Pancreatic gland characteristics, method of pancreatic transection, suture ligation of pancreatic duct, use of reinforcement, sealants, or flaps did not reduce proportion of pancreatic fistula in this multi-institutional study.

There has been recent controversy about using surgical drains after pancreatic resection. Similarly to multiple other retrospective analyses, use of surgical drains is associated with pancreatic fistula in this patient population. A recent large multi-institutional study suggested association between presence of surgical drain and pancreatic fistula after distal pancreatectomy. However, a sub-analysis suggested decreased burden of complications among patients with fistula when a surgical drain was used [17]. Retrospective data analysis is poorly suited for evaluation of risk and benefit of surgical drain after pancreatic resection. Prospective study design and analysis is required to understand the impact of surgical drains on fistula mitigation after distal pancreatectomy.

Missing data regarding pancreatic duct size and gland texture are a limitation of this study. Proportions of patients with missing pancreatic duct size and gland texture in the ACS NSQIP targeted dataset who had distal pancreatectomy approximate 60–70% and are considerably greater than the approximately 25% missing data for the same gland characteristics among patients who had pancreaticoduodenectomy. In comparison, only 110 patients (1.1% of 9577 patients) had missing pancreatic fistula data; all these patients were excluded from the study. Since pancreatic gland characteristics are most frequently extracted from the operative report, it is likely that some surgeons already doubt the usefulness of these parameters in predicting clinical outcome after distal pancreatectomy. Pancreas-specific variables in targeted dataset (such as pancreatic duct size) could be abstracted by surgical clinical reviewers from imaging reports and not operative notes; moreover, which data points are abstracted from which data source cannot be retrospectively determined.

We used two statistical approaches to address missing data. Inclusion of these missing data parameters as “unknown” into multivariable models did not have an effect on model outcomes. Further, imputation of the most likely pancreatic remnant characteristics–small pancreatic gland and soft pancreatic gland texture–did not have a significant effect on model outcomes in multivariable analysis. Patients with chronic pancreatitis, who are likely to have fibrotic parenchyma and/or pancreatic duct drainage abnormalities, were specifically excluded from this study to minimize possibility of confounding. Neither pancreatic duct size, nor gland texture were associated with pancreatic fistula after distal pancreatectomy in unadjusted, adjusted, and imputed multivariable models.

## Conclusions

Nearly 20% of patients have pancreatic fistula after distal pancreatectomy. Neither pancreatic duct size nor pancreatic gland texture are associated with pancreatic fistula after distal pancreatectomy. Potential mitigation strategies for fistula after distal pancreatectomy should focus on a combination of potential upstream pancreatic duct obstruction with or without adjunct coverage of pancreatic transection margin and not on pancreatic gland characteristics.

**Appendix A**

**Included international classification of diseases, 9th revision and 10^th^ revision diagnoses codes**

**Malignant**: 150.9, 151, 151.1, 151.2, 151.3, 151.4, 151.8, 151.9, 152, 152.8, 152.9, 153, 153.1, 153.2, 153.3, 153.5, 153.6, 153.8, 153.9, 154, 155, 155.1, 156, 156.1, 156.2, 156.8, 156.9, 157, 157.1, 157.2, 157.3, 157.4, 157.8, 157.9, 158, 158.8, 159.9, 171.5, 171.6, 171.8, 171.9, 172.9, 179, 183, 186.9, 188.9, 189, 194, 195.2, 196.2, 197.4, 197.5, 197.6, 197.7, 197.8, 198.6, 198.8, 198.89, 199.1, 200.37, 200.7, 202.8, 202.83, 202.87, 209, 209.01, 209.2, 209.25, 209.26, 209.29, 209.3, 209.41, 209.71, 209.72, 209.74, 230.2, 230.9, 258.01, C15.9, C16.1, C16.3, C16.9, C17.0, C17.8, C17.9, C18.2, C18.9, C20, C22.1, C23.0, C24.0, C24.1, C24.8, C24.9, C25.0, C25.1, C25.2, C25.3, C25.4, C25.7, C25.8, C25.9, C26.0, C48.0, C49.4, C49.9, C64.9, C78.4, C78.8, C78.89, C79.72, C79.89, C7A.010, C7A.011, C7A.095, C7A.098, C7A.1, C7A.8, C83.37, C83.39, C91.00, Z85.07, Z85.09;

**Non-malignant**: 38.9, 200.7, 209, 209.6, 209.65, 209.66, 209.69, 211, 211.1, 211.2, 211.3, 211.4, 211.5, 211.6, 211.7, 211.8, 211.9, 214.3, 215.5, 220, 227, 228, 228.1, 229.9, 230.8, 230.9, 235, 235.2, 235.3, 235.4, 235.5, 237.2, 237.3, 238.1, 238.8, 239, 239.7, 239.9, 251.2, 251.9, 255.9, 258.01, 277.89, 284.19, 287.31, 287.49, 289.59, 442.84, 456.1, 456.2, 531.5, 532.1, 532.51, 537, 537.3, 537.89, 537.9, 552.3, 553.21, 555, 557, 557.1, 557.9, 562, 562.01, 562.11, 567.9, 568.81, 568.89, 569.83, 571.5, 572.2, 572.3, 573.8, 574.1, 574.2, 575.11, 575.8, 576.1, 576.2, 576.8, 576.9, 577, 577.2, 577.8, 577.9, 592, 600, 751.69, 751.7, 782.4, 785.6, 789.32, 863.83, 996.59, 996.79, 997.41, 997.49, 998.09, 998.2, 998.59, 998.6, V57.89, K86.9, D13.6, K86.2, I72.8, K86.8, D13.9, K85.9, K86.8, A18.89, A41.9, C7B.8, C96.A, D01.49, D01.7, D12.6, D13.2, D13.5, D13.6, D13.7, D13.9, D18.03, D35.02, D36.7, D36.9, D37.2, D37.6, D37.8, D37.9, D3A.09, D3A.094, D3A.8, D48.1, D48.3, D48.9, D49, D49.0, D73.2, D73.5, D73.89, E31.21, I72.8, J44.0, K31.5, K31.7, K68.19, K83.1, K83.9, K85.2, K85.8, K85.9, K86.2, K86.3, K86.8, K86.9, M67.40, Q44.4, Q45.3, Q89.09, R19.00, R22.9, Z86.2, 790.410

## Supporting information

S1 Table Characteristics among distal pancreatectomy patients with missing duct size versus present (n = 3132). (DOCX)

S2 Table Characteristics among distal pancreatectomy patients with missing gland texture versus present (n = 3132). (DOCX)
