# Supplementary material for: Pancreatic duct size and gland texture are associated with pancreatic fistula after pancreaticoduodenectomy but not after distal pancreatectomy
Source: PLoS One. 2018 Sep 13;13(9):e0203841. doi: 10.1371/journal.pone.0203841 (PMC6136772; doi:10.1371/journal.pone.0203841)
Supplement: S1 Table — (DOCX) [file pone.0203841.s001.docx]

**Supporting Table 1: Characteristics among Distal Pancreatectomy patients with missing duct size versus present (n = 3132)**

|  | Missing (n=2424) | Present (n=743) | p-value |
| --- | --- | --- | --- |
| Age | 62 (52-71) | 65 (54-73) | 0.0004 |
| Male Sex | 1060 (43.7) | 312 (42.0) | 0.403 |
| BMI | 28.0 (24.3-32.4) | 27.2 (23.8-32.4) | 0.122 |
| Race/Ethnicity |  |  |  |
| White | 1765 (79.0) | 542 (82.5) |  |
| Black | 242 (10.8) | 67 (10.2) |  |
| Hispanic | 122 (5.5) | 27 (4.1) |  |
| Other | 104 (4.7) | 21 (3.2) | 0.153 |
| Surgical Approach |  |  |  |
| Open | 1189 (49.1) | 397 (53.4) |  |
| Minimally Invasive | 1235 (51.0) | 346 (46.6) | 0.037 |
| Malignant Diagnosis | 1346 (55.5) | 394 (53.0) | 0.231 |
| Operative time | 210 (155.5-283) | 219 (162-289) | 0.087 |
| Diabetes | 552 (22.8) | 204 (27.5) | 0.009 |
| Smoking | 391 (16.1) | 135 (18.2) | 0.191 |
| Gland Texture |  |  |  |
| Hard | 216 (8.9) | 101 (13.6) |  |
| Intermediate | 62 (2.6) | 47 (6.3) |  |
| Soft | 430 (17.7) | 374 (50.3) |  |
| Missing | 1716 (70.8) | 221 (29.7) | <0.001 |
| Pancreatic Fistula | 459 (19.2) | 147 (19.9) | 0.669 |
| Surgical Drain | 2107 (87.2) | 584 (78.6) | <0.001 |
| Percutaneous Drain | 277 (11.7) | 93 (12.7) | 0.473 |
| Peak Amylase POD#1 | 903 (104-3978) | 457 (42-2774) | <0.001 |
| Peak Amylase POD#2-30 | 162 (29-2099) | 69 (15-902) | <0.001 |
| Transfusion within 72 hrs | 331 (13.7) | 89 (12.0) | 0.238 |
| Length of Stay | 7 (5-10) | 8 (6-12) | <0.001 |
| Reoperation | 88 (3.6) | 24 (3.2) | 0.605 |
| Readmission | 418 (17.3) | 125 (16.9) | 0.781 |
| Death | 28 (1.2) | 10 (1.4) | 0.676 |

IQR: interquartile range; BMI: body mass index; POD: postoperative day

Continuous variables are expressed as median (IQR) and categorical variables are expressed as *n* (%).
