# Supplementary material for: Pancreatic duct size and gland texture are associated with pancreatic fistula after pancreaticoduodenectomy but not after distal pancreatectomy
Source: PLoS One. 2018 Sep 13;13(9):e0203841. doi: 10.1371/journal.pone.0203841 (PMC6136772; doi:10.1371/journal.pone.0203841)
Supplement: S2 Table — (DOCX) [file pone.0203841.s002.docx]

**Supporting Table 2: Characteristics among Distal Pancreatectomy patients with missing gland texture versus present (n = 3132)**

|  | Missing (n=1937) | Present (n=1230) | p-value |
| --- | --- | --- | --- |
| Age | 63 (53-72) | 63 (53-71) | 0.222 |
| Male Sex | 850 (43.9) | 522 (42.4) | 0.424 |
| BMI | 28.1 (24.4-32.4) | 27.4 (24.1-32.3) | 0.098 |
| Race/Ethnicity |  |  |  |
| White | 1384 (78.4) | 923 (82.0) |  |
| Black | 189 (10.7) | 120 (10.7) |  |
| Hispanic | 106 (6.0) | 43 (3.8) |  |
| Other | 86 (4.9) | 39 (3.5) | 0.014 |
| Surgical Approach |  |  |  |
| Open | 920 (47.5) | 666 (54.2) |  |
| Minimally Invasive | 1581 (49.9) | 564 (45.9) | <0.001 |
| Malignant Diagnosis | 1058 (54.6) | 682 (55.5) | 0.649 |
| Operative time | 209 (155-278) | 219 (161-295) | 0.011 |
| Diabetes | 467 (24.1) | 289 (23.5) | 0.693 |
| Smoking | 304 (15.7) | 222 (18.1) | 0.083 |
| Duct Size |  |  |  |
| <3 mm | 113 (5.8) | 315 (25.6) |  |
| 3-6 mm | 67 (3.5) | 150 (12.2) |  |
| >6 mm | 41 (2.1) | 57 (4.6) |  |
| Missing | 1716 (88.6) | 708 (57.6) | <0.001 |
| Pancreatic Fistula | 372 (19.5) | 234 (19.2) | 0.863 |
| Surgical Drain | 1688 (87.4) | 1003 (81.7) | <0.001 |
| Percutaneous Drain | 230 (12.2) | 140 (11.6) | 0.601 |
| Peak Amylase POD#1 | 701 (73-3588) | 536 (47-3011) | 0.089 |
| Peak Amylase POD#2-30 | 137 (25.5-1729) | 80 (16-1028) | <0.001 |
| Transfusion within 72 hrs | 272 (14.0) | 148 (12.0) | 0.104 |
| Length of Stay | 7 (5-11) | 8 (6-12) | <0.001 |
| Reoperation | 74 (3.8) | 38 (3.1) | 0.278 |
| Readmission | 349 (18.1) | 194 (15.8) | 0.103 |
| Death | 19 (0.98) | 19 (1.5) | 0.156 |

IQR: interquartile range; BMI: body mass index; POD: postoperative day

Continuous variables are expressed as median (IQR) and categorical variables are expressed as *n* (%).
